# High Cytotoxic Activity of Phosphonium Salts and Their Complementary Selectivity towards HeLa and K562 Cancer Cells: Identification of Tri-*n*-butyl-*n*-hexadecylphosphonium bromide as a Highly Potent Anti-HeLa Phosphonium Salt

**DOI:** 10.1002/open.201100003

**Published:** 2012-02-17

**Authors:** Barbara Bachowska, Julia Kazmierczak-Baranska, Marcin Cieslak, Barbara Nawrot, Dorota Szczęsna, Joanna Skalik, Piotr Bałczewski

**Affiliations:** [a] Institute of Chemistry, Environmental Protection and Biotechnology, Jan Długosz University in CzęstochowaArmii Krajowej 13/15, 42-200 Częstochowa (Poland) E-mail: pbalczew@bilbo.cbmm.lodz.pl; [b] Department of Bioorganic Chemistry, Centre of Molecular and Macromolecular Studies, Polish Academy of SciencesSienkiewicza 112, 90-363 Łódź (Poland); [c] Department of Heteroorganic Chemistry, Centre of Molecular and Macromolecular Studies, Polish Academy of SciencesSienkiewicza 112, 90-363 Łódź (Poland)

**Keywords:** phosphonium cations, cytotoxicity, gene delivery, cancer cells, transfection

## Abstract

Quaternary ammonium and phosphonium salts have been screened for their toxic effect on HeLa and K562 cancer cell lines, as well as on normal HUVEC cells. Tri-*n*-butyl-*n*-hexadecylphosphonium bromide, the first phosphonium salt with a halogen anion tested against HeLa cells, was 12 times more potent (IC_50_ <5 μm after 24 and 48 h) than the clinically used reference compound cisplatin and 17 times more potent than tri-*n*-hexyltetradecylphosphonium bis(trifluoromethylsulfonyl)imide, which, to the best of our knowledge, is the first phosphonium salt to be evaluated in HeLa cells. However, it was inactive against K562 cells (24 and 48 h). According to a caspase-3/7 assay, its toxicity has not been connected with the induction of apoptosis. In contrast, triphenylalkylphosphonium iodides with shorter C_1–5_ alkyl chains were inactive against HeLa cells but very active against K562 cells (IC_50_=6–10 μm after 48 h). Phosphonium cations with halide counterions proved to be more potent than those with (CF_3_SO_2_)_2_N^−^ as the anion, as in the anticancer agent NSC 747251, or other anions in molecules with similar alkyl chain lengths. On the other hand, a series of ammonium salts containing a short methylthiomethyl or methoxymethyl side chain revealed low cytotoxicity (IC_50_ >500 μm after 24 and 48 h) against both HeLa and K562 cancer cell lines as well as normal HUVEC cells, showing that the nontoxic N^+^CH_2_YMe (Y=S, O) structural motif in ammonium salts could be suitable for further optimization and development, especially in transfection experiments.

## Introduction

In recent decades, quaternary heteronium salts (QHSs) with its positive charge localized or delocalized on heteroatoms, such as nitrogen, phosphorus or sulfur, have found various applications as reagents, bio-, organo- and metallocatalysts, green solvents, biologically active compounds, and new materials. Among this group of QHS compounds, ionic liquids (ILs) with melting points below 100 °C have become the subject of a great number of studies performed both in academia and industry.^1^–^3^ As a consequence of their diverse utility and propagation in the natural environment, various toxicological investigations, including eco-phyto-toxicological, microbiological and cytotoxicological studies, have been carried out.^4^–^9^

In medical and pharmaceutical research, QHS derivatives are termed cationic lipophilic salts, delocalized lipophilic cations, or simply lipophilic cations. Due to the difference in trans-mitochondrial negative electric membrane potentials between carcinoma and normal cells, these compounds selectively accumulate in the mitochondria of carcinoma cells, particularly those of human origin.^10^–^13^ This in vitro selectivity has been found to range from 10- to 100-fold.^14^ Therefore, numerous cytotoxicological investigations have been devoted to various lipophilic QHSs, including rhodamine 123, dequalinium chloride, victoria blue BO, safranin O, and various phosphonium salts, as selective, anti-carcinoma chemotherapeutic agents.^14^, ^15^

More recently, the interest in biological studies on QHSs has increased, particularly, towards the assessment of the risk of ILs to humans and the environment. The HeLa cell line^9^ and the two colon carcinoma cell lines, HT-29 and CaCo-2,^16^ have been used to evaluate toxicity of various nitrogen-containing ILs involving different classes of cations. The CaCo-2 cell line has also been used in evaluation of the toxicity of imidazolium, guanidinium, ammonium, phosphonium, pyridinium, and pyrrolidinium cations,^16^, ^17^ and showed that increasing the length of the substituent chain contributed to a significant increase in toxicity. In addition, the cytotoxicity of these compounds was strongly affected by the type of counterion. The effects of different head groups and functionalized side chains on the cytotoxicity of ILs have been described in other investigations.^7^ Polycationic phosphorus dendrimers bearing various types of protonated amine terminal groups (e.g., pyrrolidine, morpholine, methyl piperazine, or phenyl piperazine) have previously been assayed to determine their cytotoxicity, which was found to be weak against three cell lines, HUVEC, HEK293 and HeLa, the latter two being cancerous.^18^ Finally, a quantum-chemical-based guide to analyze cytotoxicity of ILs was also introduced in very recent studies.^4^

**Figure 1:**
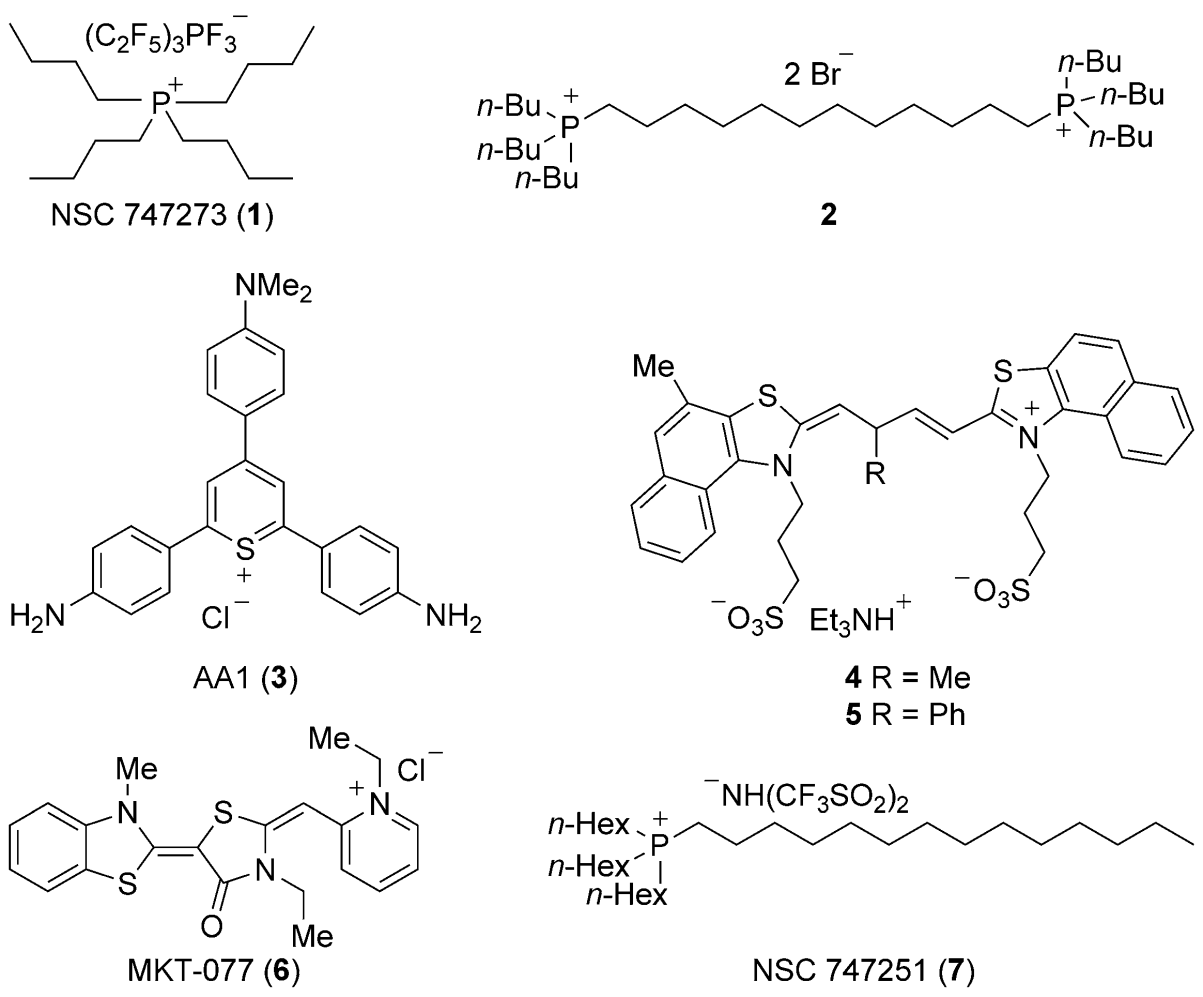
Phosphorus- and sulfur-containing lipophilic QHS.

The biological activity of phosphonium salts is known, and it is sometimes compared with the activity of analogous ammonium salts.^19^ However, in a number of such investigations, the activity was very low. Kumar and Malhotra^20^ investigated the cytotoxicity of phosphonium salts containing 4–14 carbon-atom chains and PF_6_^−^, N(CF_3_SO_2_)_2_^−^, BF_4_^−^, or [PF_3_(C_2_F_5_)_3_]^−^ anions, on a set of human tumor cell lines, not including HeLa cells (Figure 1). They observed that phosphonium salts were more active and less cytotoxic than ammonium salts. Other reports noted that a change from an ammonium to a phosphonium cation elicited no change in inhibitory potential.

Bisphosphonic salts with C_12_-chains, such as 1,12-bis(tributylphosphonium)dodecyl dibromide **2** (Figure 1), exhibited moderate phospholamban (PLB) inhibition and antifungal activity.^21^ Delikatny et al. investigated the effect of a triphenyl-containing salt, and tris(4-dimethylaminophenyl) mono- and bisphosphonium salts with short carbon chains on breast cancer cells, DU-4475 and HBL-100, and the triphenyl derivative exhibited selective growth inhibition.^15^

Among cationic, lipophilic, sulfur-containing salts, the correlation between the number and character of sulfur atoms, and biological activity remains unknown. The cyclic organosulfur compounds explored so far include sulfonium and bivalent sulfur atoms. For example, the thiopyrylium salt AA1 (**3**)^14^ strongly inhibited mitochondrial ATPase activity and proved to be 10-times more toxic to colon carcinoma cells (CX-1) than to normal monkey kidney cells (CV-1). Thiacarbocyanine dyes (e.g., **4** and **5**, Figure 1) exhibited cytotoxicity in human colon carcinoma cells and additionally inhibited bovine heart mitochondrial NADH-ubiquinone reductase activity.^22^ Other representatives of the cyanines showed cytotoxicity that in part correlated with the length of alkyl chains.^23^ MKT-077 (**6**, Figure 1), formerly known as FJ-776, exhibited a significant antitumor activity in a variety of model systems and was tested in clinical trials.^24^–^26^ MKT-077 was found to bind to the hsp70 family member, mortalin (mot-2), and to abrogated its interactions with the tumor suppressor protein p53.^27^

**Scheme 1:**
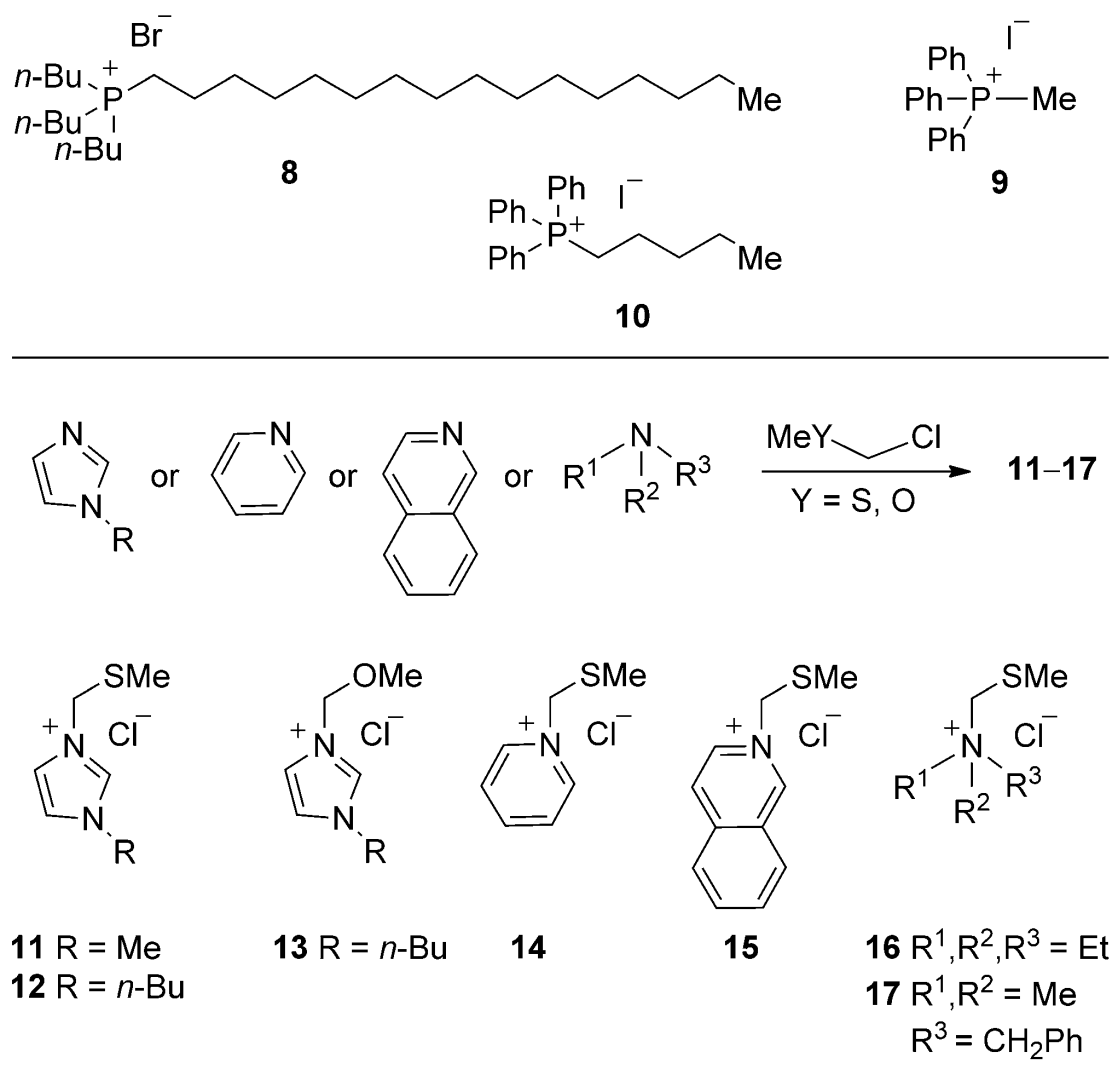
Phosphonium 8–10 and methylthiomethyl and methoxymethyl ammonium substituted QHS 11–17.

Initially, we focused on the cytotoxic activity of three phosphonium halides (**8**–**10**, Scheme 1), possessing tri-*n*-butylphosphonium and triphenylphosphonium cations in addition to long and short alkyl chains, on human cervix carcinoma (HeLa) and human chronic myelogenous leukemia (K562) cells. We then synthesized a series of new QHS cations **11**–**17** (Scheme 1) containing an acyclic, bivalent sulfur atom in a short methylthiomethyl chain. These compounds were screened for their cytotoxic activity towards two cancer cell lines (HeLa and K562) and noncancerous HUVEC cells. We also evaluated their properties as DNA carriers in transfection of mammalian cells.

## Results and Discussion

### Synthesis of phosphonium and ammonium salts

Ammonium salts **11**–**17** were prepared from 1-methylimidazole, 1-*n*-butylimidazole, pyridine, isoquinoline and two aliphatic amines (i.e., triethylamine and benzyldimethylamine). These reagents were reacted with methylthiomethyl chloride (for **11, 12, 14**–**17**) or methoxymethyl chloride (for **13**). 1-*n*-Butylimidazole derivative **13** in which the sulfur atom is replaced by oxygen was synthesized as a reference compound. Methylthiomethyl chloride was obtained using a Pummerer reaction of dimethyl sulfoxide with 95 % excess of thionyl chloride in dichlormethane in >90 % yield (m.p. 104–106 °C).^28^ This reaction was also performed in chloroform, using only a 1.22–1.50-fold excess of thionyl chloride. However, under these conditions, the product was obtained in lower yields (62 %). Products **11**–**17** possess hydrophilic ammonium groups and showed amphiphilic character, despite the short hydrophobic hydrocarbon chain, MeYCH_2_ (Y=S, O). All the salts were soluble in water and methanol, and partly soluble in chloroform and DMSO. None of them were soluble in hexane, ethyl acetate or acetone, except compound **13** which was soluble in acetone.

### Cytotoxic properties of compounds 8–17 towards HeLa and K562 cells

The cytotoxicity of test compounds **8**–**17** was measured using a 3-(4,5-dimethylthiazol-2-yl)-2,5-diphenyltetrazolium bromide (MTT) assay, and the results are summarized in Table 1. The results show that the cytotoxicity of phosphonium salts **8**–**10** is much more potent than that of ammonium salts **11**–**17** against HeLa, K562 and HUVEC cells. It is known that phosphonium salts, as cationic lipophilic compounds, selectively accumulate in mitochondria of neoplastic cells.^15^, ^29^

**Table 1:** IC_50_ (μM) values calculated for compounds 8–17 from the dose–response curves in HeLa, K562 and HUVEC cells. cisplatin was used as a reference compound. IC_50_ values represent the mean (±SD) of eight or four, in the case of compounds 9 and 10, replicate cultures.

Compd	HeLa		K562		HUVEC	
	24 h	48 h	24 h	48 h	24 h	48 h
**8**	4.7±2.2	4.8±7.1^[a]^	400±200	500±50	3.75±1.3	0.325±0.082
**9**	600±42	100±5	900±54	10±0.5	–	–
**10**	90±3.6	60±3	50±3.5	6±0.3	–	–
**11**–**17**	>500	>500	>500	>500	>500	>500
cisplatin	55±9^[b]^	25±5.4^[b]^	200±40^[b]^	150±30^[b]^	140^[c]^	95±43^[c]^

[a] Compound **8** precipitated in the cell culture medium when used at a concentration of 500 μm. Therefore this data point was omitted [b] Cisplatin was freshly dissolved in DMSO. [c] Cisplatin was dissolved in water.

Tri-*n*-butyl-*n*-hexadecylphosphonium bromide **8**, as a phosphonium salt with a halogen anion and balanced lengths of C_4_ and C_16_ alkyl chains, was tested in HeLa cells. After a 24 h incubation period, this compound showed an IC_50_ value of approximately 5 μm, while cisplatin, used as a reference compound, had an IC_50_ value of 55 μm. This means that **8** is roughly 10-times more potent in terms of cytotoxicity than cisplatin under the same experimental conditions. While the toxicity of **8** did not change after a longer incubation time of 48 h, the toxicity of cisplatin seemed to increase, since a lower IC_50_ value of 25 μm versus 55 μm was measured after 48 h. Nevertheless, the toxicity of **8** against cancer cells was still superior to that of cisplatin. Moreover, compound **8** was found to be 17-times more potent than NSC 747251 (**7**, Figure 1)^6^ under the same assay conditions. Currently, compound **7** is the only phosphonium salt to have been evaluated in HeLa cells. Phosphonium salt **8** was approximately 100-times more toxic against HeLa cells than ammonium salts **11**–**17**, but remained inactive against K562 cells after 24 and 48 h incubation. Compound **8** has also been used as an inhibitor of the dynamine GTPase activity,^30^ an antifungal agent with broad-spectrum activity^19^ and a phase-transfer catalyst.^31^. Bromide **8** was also tested for its ability to induce apoptosis in HeLa cells using a caspase-3/7 assay (see section below).

Triphenylalkylphosphonium iodides **9** and **10** with shorter C_1_ and C_5_ alkyl chains, respectively, also showed complementary activity, being inactive against HeLa cells, but very active against K562 cells (IC_50_=6–10 μm after 48 h). Neither salt has previously been evaluated against HeLa and K562 cells. Iodide **9** has only previously been screened against Ehrlich ascites carcinoma (EAC), lymphoid leukemia (LI210) and human epidermoid carcinoma of the nasopharynx (KB cell line).^31^ Iodide **10** has previously been screened against influenza type A.^32^ In contrast, screened ammonium compounds **11**–**17**, possessing a short heteroatom N^+^CH_2_SMe chain, showed IC_50_ values greater than 500 μm in experiments performed after either 24 or 48 h incubation, a concentration which is characteristic for compounds of rather low toxicity.

### Evaluation of compounds 11–17 as DNA carriers

Although a series of chemical carriers had previously been synthesized, most of them remain restricted to in vivo gene therapy, due to toxicity and low transfection efficiencies. Thus, the results discussed above, where compounds **11**–**17** show a lack of toxicity (Table 1), provided a good opportunity to test our ammonium salts as gene delivery vehicles, since they could electrostatically interact with negatively charged DNA or RNA molecules.

**Figure 2:**
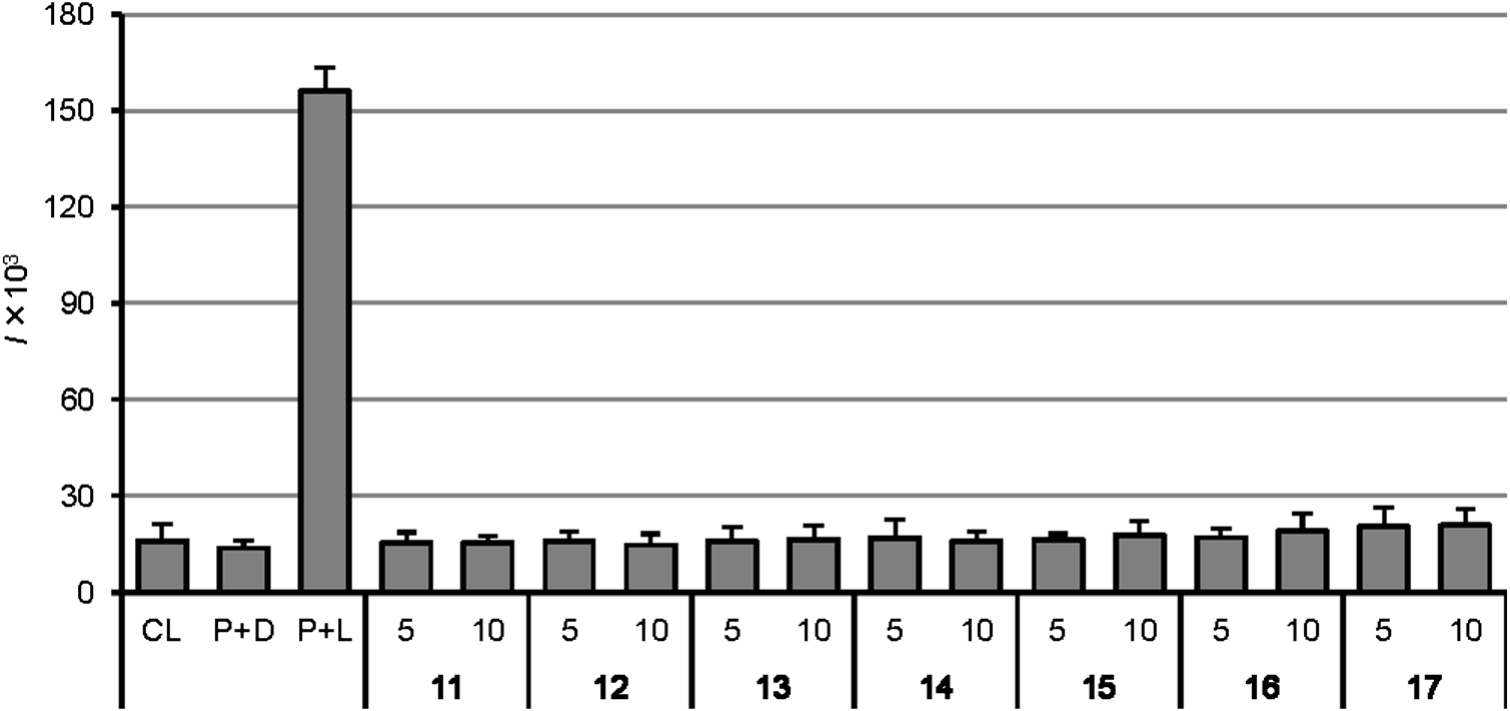
Fluorescence intensity (*I*) showing the expression of GFP in HEK293T cells transfected with p_max_GFP plasmid DNA in the presence of compounds 11–17 either in 5- or 10-fold charge excess above the total charge of DNA. Cells treated with lipofectamine only (CL), cells incubated with plasmid DNA only (P+D), cells transfected with p_max_GFP vector DNA in the presence of lipofectamine (P+L). Error bars indicate standard deviations (±SD).

To test this potential application, HEK293T cells were incubated with the plasmid p_max_GFP encoding enhanced green fluorescence protein (EGFP) in the presence of compounds **11**–**17** at a charge ratio (compound/DNA) of 5:1 and 10:1. After an incubation time of 48 h, the transfection efficiency of compounds **11**–**17** was determined by measuring the level of expressed EGFP in transfected cells. Cells treated with lipofectamine only (CL) or p_max_GFP only (P+D) were used as negative controls, while cells transfected with p_max_GFP in the presence of lipofectamine (P+L) were used as positive controls. As shown in Figure 2, the expression of EGFP was low in cells grown in the presence of either p_max_GFP (P+D) or lipofectamine (CL) alone. On the other hand, EGFP expression dramatically increased in cells transfected with p_max_GFP in the presence of lipofectamine (P+L). Unfortunately, no significant increase in EGFP expression was observed in the presence of compounds **11**–**17**, used at a charge ratio (compound/DNA) of 5:1 and 10:1. Therefore, we concluded that these compounds do not facilitate DNA delivery into HEK293T cells. A further increase in lipophilicity of cationic moieties should improve the gene delivery properties of the screened compounds.^33^ Lengthening the alkyl side chains usually improves lipophilicity, however, it could also cause an increase in cytotoxicity.^7^

### Caspase 3 and 7 activity in HeLa cells treated with compound 8

**Figure 3:**
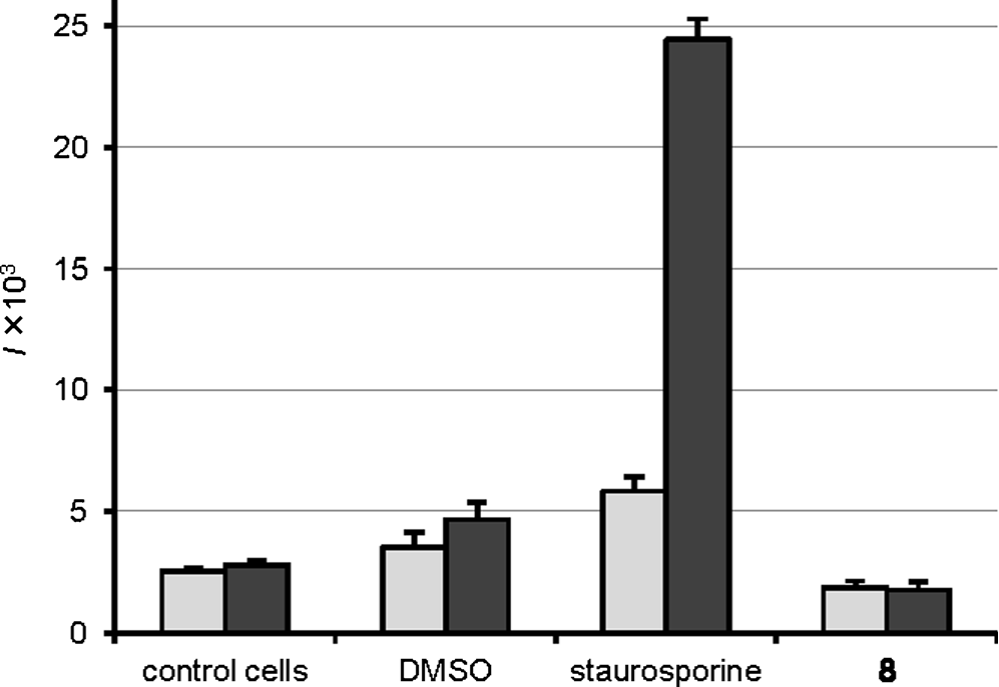
Fluorescence intensity (*I*) showing apoptosis of HeLa cells treated with compound 8 after 6 h (▪) and 24 h (▪). Apoptosis was determined using a caspase-3/7 assay. Untreated cells (control cells), cells treated with 2 % DMSO (DMSO), cells treated with staurosporine (staurosporine), cells treated with 8 dissolved in aqueous 2 % DMSO (8). Error bars indicate standard deviations (±SD) of four replicates.

Caspases 3 and 7, members of the cysteine aspartic acid-specific protease family, play key effector roles in apoptosis in mammalian cells.^34^ These enzymes are responsible for the cleavage of selected cellular proteins, such as cytoskeletal proteins, which leads to the typical morphological changes observed in cells undergoing apoptosis. Increased activity of caspases 3 and 7 is an indicator of apoptosis. Our experiments have shown that the level of caspase 3 and 7 activity in cells treated with **8** was not increased compared to control cells or cells treated with DMSO (Figure 3). In contrast, an increase of activity of caspase 3 and 7 was observed in cells treated with staurosporine, a compound known to induce apoptosis. This result indicates that the toxicity of **8** does not result from induction of apoptosis, but rather originates from another pathway.

## Conclusion

In summary, some quaternary heteronium (P, S) halides (Cl, Br, I) have been investigated for their cytotoxicity against HeLa and K562 cancer cells. Alkyl-tri-*n*-butylphosphonium and alkyltriphenylphosphonium halides (Br, I), possessing different lengths of alkyl chains (C_1–16_), were found to be very potent cytotoxic compounds against HeLa and K562 cells, but showed a complementary activity. Phosphonium bromide **8** proved to be several times more potent than the clinically used reference drug cisplatin. Moreover, **8** is the most active phosphonium salt against on HeLa cells, known so far. On K562 cells, iodides **9** and **10** were also several times more potent than cisplatin. Therefore, these compounds could be regarded as potential anticancer drugs in targeted therapies.^35^ On the other hand, another family of quaternary heteronium salts, *N*-methylthiomethyl-substituted ammonium halides, were found to be nontoxic against HeLa, K562 and HUVEC cells. Optimization investigations are currently in progress in our collaborative laboratories directed towards further screening of new phosphonium salts based on the nontoxic N^+^CH_2_SMe structural motif, found to improve lipophilicity for gene transfection.

## Experimental Section

### Synthesis of 8–17

**General**: The ^1^H NMR (200 MHz) and ^13^C NMR (50 MHz) spectra were recorded on a Brucker AV 200 spectrometer. The mass spectra of pure compounds were obtained using a Finigan Mat 95 mass spectrometer.

**Tri-*n*-butyl-*n*-hexadecylphosphonium bromide (8)**: Equimolar amounts of 1-bromo-*n*-hexadecane and tri-*n*-butylphosphine were heated to 65 °C for 3 d. Phosphonium bromide **8** was isolated as a white solid (60–70 %):^36^ mp: 56–58 °C; ^1^H NMR (200 MHz, CDCl_3_, 25 °C): δ=0.81–0.98 (m, 12 H, 4CH_3_), 1.22 (br s, 22 H, 11CH_2_), 1.31–1.53 (m, 18 H, 9CH_2_), 2.36–2.50 ppm (m, 8 H, 4CH_2_); ^13^C NMR (50 MHz, CDCl_3_, 25 °C): δ=12.6 (s, 3CH_3_), 13.0 (s, CH_3_), 18.6 (d, ^1^*J*_C–P_=47.3 Hz, P(CH_2_*n*-Pr)_3_), 18.8 (d, ^1^*J*_C–P_=47.0 Hz, P*C*H_2_(CH_2_)_14_CH_3_); 21.4 (d, ^3^*J*_C–P_=4.5 Hz, P(CH_2_)_2_*C*H_2_(CH_2_)_12_CH_3_), 22.1 (s, *C*H_2_), 23.3 (s, *mC*H_2_), 23.5 (d, ^2^*J*_C–P_=11.6 Hz, P(CH_2_*C*H_2_)_3_), 28.4 (s, CH_2_), 28.8 (s, *n*CH_2_), 29.0 (s, CH_2_), 29.1 (br s, *r*CH_2_), 30.3 (d, ^2^*J*_C–P_=14.6 Hz, PCH_2_CH_2_(CH_2_)_13_CH_3_), 31.3 ppm (s, CH_2_) (where *m*+*n*+*r*=11C); MS (FAB+, Cs^+^,13 keV): *m/z* (%): 427 (100, C_16_H_33_P-*n*Bu_3_) [*M*]^+^; MS (FAB−, Cs^+^,13 keV): *m/z* (%): 585 (7) [*M*+2Br]^−^, 587 (3) [*M*+2Br]^−^, 589 (7, 13, 7) [*M*+2Br]^−^, 222 (85), 79 (100) [*M*+Br]^−^, 81 (100) [*M*+Br]^−^; HRMS (EI): *m/z* [*M*]^+^ calcd for C_28_H_60_P: 427.44327, found: 427.44344; Anal. calcd for C_28_H_60_BrP: C 66.27, H 11.83, P 6.11, found: C 66.32, H 11.98, P 6.17.

Phosphonium iodides **9** and **10** were synthesized accordingly from triphenyl phosphine and methyl iodide or *n*-pentyl iodide, respectively, as previously described.^37^, ^38^

**1-*n*-Butyl-3-methoxymethylimidazolium chloride (13):** Methoxymethyl chloride (0.16 g, 0.15 mL, 2.01 mmol) was added to a stirred solution of 1-*n*-butylimidazole (0.25 g, 0.26 mL, 2.01 mmol), and the resulting mixture was stirred for 1 h at RT. Salt **13** was obtained as a yellow oil (0.41 g, 100 %): ^1^H NMR (200 MHz, CDCl_3_, 25 °C): *δ*=0.80 (t, 3 H, ^3^*J*_H–H_=7.4 Hz, CH_3_), 1.23 (q, 2 H, ^3^*J*_H–H_=7.4 Hz, C*H*_2_CH_3_), 1.79 (dt, 2 H, ^3^*J*_H–H_=7.4 Hz, CH_2_C*H*_2_CH_3_), 3.30 (s, 3 H, OCH_3_), 4.24 (t, 2 H, ^3^*J*_H–H_=7.3 Hz, NCH_2_), 5.65 (s, 2 H, CH_2_O), 7.63 (s, 2 H, *H*C=C*H*), 10.64 ppm (s, 1 H, N^+^=CH); ^13^C NMR (50 MHz, CDCl_3_, 25 °C): *δ*=12.1 (s, *C*H_3_CH_2_), 18.0 (s, O*C*H_3_), 30.6 (s, CH_3_*C*H_2_), 48.5 (s, CH_3_*C*H_2_CH_2_), 56.2 (s, N*C*H_2_), 78.6 (s, N^+^*C*H_2_O), 120.7 (s, N*C*H=CH), 121.6 (s, N^+^*C*H=CH), 134.5 ppm (s, N*C*H=N^+^); MS (FAB+,Cs^+^, 13 keV): *m/z* (%): 373 (7) [2*M*+Cl]^+^, 169 (100) [*M*]^+^; MS (FAB−, Cs^+^, 13 keV): *m/z* (%): 443 (15) [2*M*+3Cl]^−^, 339 (18), 241 (62), 239 (100) [*M*+2Cl]^−^, 135 (76), 133 (68), 127 (70); HRMS (EI): *m/z* [*M*]^+^ calcd for C_9_H_17_N_2_O: 169.13409, found: 169.13366; Anal. calcd for C_9_H_17_ClN_2_O: C 52.76, H 8.37, N 13.68, found: C 52.38, H 8.72, N 13.96.

The synthesis of compound **16** (below) is given as a representative example for the synthesis of compounds **11**, **12**, **14**–**17**.

**Triethyl(methylthiomethyl)ammonium chloride (16)**: Under Ar, methylthiomethyl chloride (1.93 g, 20 mmol) was added to a stirred solution of triethylamine (2.02 g, 20 mmol), and the resulting mixture was stirred for 72 h at RT. Salt **16** was obtained as a beige solid (3.95 g, 100 %): mp: 101–102 °C; ^1^H NMR (200 MHz, CDCl_3_, 25 °C): *δ*=1.41 (t, 9 H, ^3^*J*_H–H_=7.1, CH_2_C*H*_3_), 2.51 (s, 3 H, SCH_3_), 3.53 (q, 6 H, ^3^*J*_H–H_=7.1, C*H*_2_CH_3_), 5.06 (s, 2 H, N^+^CH_2_S); ^13^C NMR (50 MHz, CDCl_3_, 25 °C): *δ*=8.2 (s, N^+^CH_2_*C*H_3_), 18.0 (s, S*C*H_3_), 52.6 (s, N^+^*C*H_2_CH_3_), 64.2 (s, N^+^*C*H_2_S); MS (CI): *m/z* (%): 162 (100) [Et_3_N+CH_2_SCH_3_]^+^; HRMS (CI): *m/z* [*M*]^+^ calcd for C_8_H_20_NS: 162.13165, found: 162.13213; Anal. calcd for C_8_H_20_ClNS: C 48.54, H 10.11, S 16.18, N 7.08, found: C 48.31, H 10.02, S 15.93, N 7.10.

### Biology

*MTT assay*: The cytotoxicity of compounds **8**–**17** was measured using a 3-(4,5-dimethylthiazol-2-yl)-2,5-diphenyltetrazolium bromide (MTT) assay. This assay relies on the reduction of MTT by succinate dehydrogenase, intervening in the respiratory mitochondrial chain of viable cells. The water-soluble yellow MTT is converted to water-insoluble purple formazan, which is then dissolved in organic solvents and measured using a spectrophotometer. The percentage of living cells is calculated from the corrected formazan absorbance. HeLa, K562 or HUVEC cells (5×10^3^ per well) were seeded in a 96-well plate (Nunc, Penfield, NY, USA). After a 24 h incubation period, the cells were exposed to the test compounds for 24 or 48 h. The cytotoxicity of each compound was tested at four concentrations: 50 nm, 500 nm, 50 μm and 500 μm. Compounds **9** and **10** were tested at 1 nm, 100 nm, 10 μm and 1 mm. Cells grown in the presence of 2 % DMSO served as a control (100 % viability). After 24 or 48 h incubation with the test compounds, the MTT reagent was added and incubation was continued for 2 h. The formazan crystals were dissolved in 20 % sodium dodecyl sulfate (SDS) and 50 % DMF (pH 4.7).The absorbance was measured at 570 and 630 nm using an ELx800 microplate reader (BioTek, Winooski, VT, USA). Based on the dose–response curves, IC_50_ values were calculated and represent the mean ±SD of eight (for compound **9**) or four (for compound **10**) replicate cultures.

*Transfection evaluation*: HEK293T (human embryonic kidney) grown in Dulbecco′s modified Eagle′s medium (DMEM) supplemented with 10 % fetal bovine serum (FBS) were transferred to a 96-well plate in 100 μL aliquots 24 h before transfection (2×10^4^ cells per well). The transfection procedure for a single 96-well plate was as follows. OPTI-MEM (25 μL) was mixed with Lipofectamine 2000 (0.1 μL, Invitrogen, CA, USA). In parallel, OPTI-MEM (25 μL) was mixed with 0.05 μg p_max_GFP vector DNA (50 ng per well; Lonza, Basel, Switzerland). After a 5 min incubation period at room temperature, both solutions were combined resulting in a Lipofectamine–DNA ratio of 2:1 (*v*/*w*) and incubated for an additional 20 min. The mixture was then added to a 96-well plate as a positive control. For tested compounds, the transfection procedure was performed in the same way as described for lipofectamine, except that the amount of tested compound was adjusted to achieve a charge ratio of 5:1 and 10:1 (compound/DNA). Transfection mixtures containing either DNA plasmid or lipofectamine only were used as negative controls. Each transfection experiment was repeated in four separate wells. After an additional 48 h incubation at 37 °C in a 5 % CO_2_ atmosphere, the culture medium was removed, and the cells were lysed for 30 min in phosphate-buffered saline (PBS) containing 1 % Triton X-100 (200 μL per well, pH 7.4). Fluorescence was measured with a FLUOstar Omega plate reader (BMG Labtech, Offenburg, Germany), with excitation and emission filters of 485/20 nm and 520/20 nm, respectively.

*Caspase-3/7 assay*: HeLa cells were cultured in RPMI 1640 medium supplemented with antibiotics and 10 % fetal calf serum (FCS) at 37 °C in a 5 % CO_2_ atmosphere. Cells (15×10^3^) were seeded in each well of a 96-well plate. After 24 h, cells were exposed to test compound at a concentration of four-times the IC_50_ value and incubated for another 24 h. As controls, cells were exposed to DMSO only (2 % final concentration), staurosporine (1 μm, Sigma, St. Louis, MO, USA) and cisplatin (70 μm, Sigma, St. Louis, MO, USA). The induction of cell apoptosis was analyzed using the Apo-ONE homogeneous caspase-3/7 assay (Promega, Madison, WI, USA). After 24 h of incubation with the test compounds, cells were treated with the caspase-3/7 reagent (according to manufacturer's instructions) and incubated for an additional hour at room temperature. The fluorescence intensity was measured using a FLUOstar Omega plate reader (BMG Labtech, Offenburg, Germany), with excitation and emission filters of 485/20 nm and 520/20 nm, respectively. Untreated cells and cells treated with staurosporine were used as negative and positive controls, respectively.
